# *De novo* CNVs in bipolar affective disorder and schizophrenia

**DOI:** 10.1093/hmg/ddu379

**Published:** 2014-07-23

**Authors:** Lyudmila Georgieva, Elliott Rees, Jennifer L. Moran, Kimberly D. Chambert, Vihra Milanova, Nicholas Craddock, Shaun Purcell, Pamela Sklar, Steven McCarroll, Peter Holmans, Michael C. O'Donovan, Michael J. Owen, George Kirov

**Affiliations:** 1 Medical Research Council Centre for Neuropsychiatric Genetics and Genomics, Institute of Psychological Medicine and Clinical Neurosciences, Cardiff University, Cardiff, UK,; 2 Stanley Center for Psychiatric Research, Broad Institute of MIT and Harvard, Cambridge, MA, USA,; 3 Department of Psychiatry, Medical University, Sofia, Bulgaria,; 4 Division of Psychiatric Genomics, Department of Psychiatry, Mount Sinai School of Medicine, New York, NY, USA and; 5 Psychiatric and Neurodevelopmental Genetics Unit, Massachusetts General Hospital, Boston, MA, USA

## Abstract

An increased rate of *de novo* copy number variants (CNVs) has been found in schizophrenia (SZ), autism and developmental delay. An increased rate has also been reported in bipolar affective disorder (BD). Here, in a larger BD sample, we aimed to replicate these findings and compare *de novo* CNVs between SZ and BD. We used Illumina microarrays to genotype 368 BD probands, 76 SZ probands and all their parents. Copy number variants were called by PennCNV and filtered for frequency (<1%) and size (>10 kb). Putative *de novo* CNVs were validated with the z-score algorithm, manual inspection of log R ratios (LRR) and qPCR probes. We found 15 *de novo* CNVs in BD (4.1% rate) and 6 in SZ (7.9% rate). Combining results with previous studies and using a cut-off of >100 kb, the rate of *de novo* CNVs in BD was intermediate between controls and SZ: 1.5% in controls, 2.2% in BD and 4.3% in SZ. Only the differences between SZ and BD and SZ and controls were significant. The median size of *de novo* CNVs in BD (448 kb) was also intermediate between SZ (613 kb) and controls (338 kb), but only the comparison between SZ and controls was significant. Only one *de novo* CNV in BD was in a confirmed SZ locus (16p11.2). Sporadic or early onset cases were not more likely to have *de novo* CNVs. We conclude that *de novo* CNVs play a smaller role in BD compared with SZ. Patients with a positive family history can also harbour *de novo* mutations.

## INTRODUCTION

Bipolar affective disorder (BD) has a life-time risk of ∼1% in the general population and a 10-fold increased risk in first-degree relatives (1). The heritability estimates range between 59 and 87% (2–4). It is a complex genetic disorder, with a high degree of genetic and phenotypic heterogeneity (5). Genome-wide association studies based on common genetic variants have identified a number of loci at compelling levels of statistical support (6–10). It has been estimated that about a third of the genetic variance in risk is contributed by common alleles that are tagged by SNPs on genotyping arrays (11).

Rare, moderate to highly penetrant copy number variants (CNVs) have been clearly established as risk factors for several neuropsychiatric disorders: schizophrenia (SZ), autism spectrum disorder and intellectual disability/developmental delay (ID/DD) (12–16). Therefore, CNVs may also account for some of the unexplained heritability of BD. Studies on BD have yielded conflicting results, with modest enrichments for CNVs in some studies but not in others (17–22). An enrichment of *de novo* CNVs in individuals with BD was first reported by Malhotra *et al*. (21) This team identified 10 CNVs in 185 probands, a rate of 5.4% (or 4.3% rate per person, as 2 probands had 2 CNVs each), compared with 4 CNVs in 426 controls (a rate of 0.9%) suggesting that this class of variants is involved in the disorder, particularly in early-onset cases. More recently, Noor *et al*. (23) found 8 *de novo* CNVs among 215 BD probands, a rate of 3.7%. No new control group was tested in that study, but the rate was considered increased compared with control rates from previous studies of 1–2%.

We aimed to investigate the role of *de novo* CNVs in the aetiology of BD in the largest sample of BD parent-proband trios tested to date and compare them with *de novo* CNVs found in SZ patients.

## RESULTS

**Figure 1.**
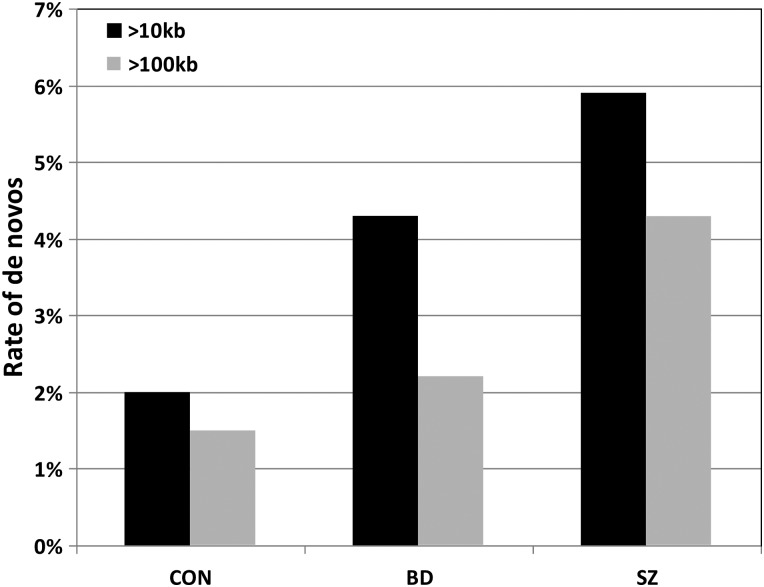
Comparison of the *de novo* rates for CNVs >10 and >100 kb in controls (CON), BD and SZ, based on the studies listed in Table 2.

We genotyped 368 BD and 76 SZ probands and all their parents [after quality control (QC) filtering]. We successfully validated 21 *de novo* CNVs: 13 deletions and 8 duplications (Table 1). Of those, 15 were found in BD probands, a rate of 4.1% (2.4% for CNVs >100 kb) and 6 in SZ probands, a rate of 7.9% (6.4% for CNVs >100 kb). These differences were not significant, but the current sample sizes were clearly too small. One BD proband had two *de novo* CNVs, so the *de novo* can also be expressed as affecting 14 of 368 (3.8%) probands. We then analysed the current data together with those from four previous *de novo* CNV studies in BD and/or SZ (Table 2). In order to be conservative in the analysis, we restricted the data to large CNVs (>100 kb), as smaller ones might not have been called with some of the arrays used in the previous studies. Using the >100-kb cut-off, the *de novo* rates were 1.5, 2.2 and 4.3% in controls, BD and SZ, respectively (Fig. 1 and Table 2). The differences between SZ and BD and between SZ and controls were significant (*P* = 0.015 and 4.3 × 10^−7^, respectively); however, the increase in the *de novo* rate in BD over controls was not significant (*P* = 0.21, Table 2). For completion, we also present the results for CNVs of >10 kb (Table 2 and Fig. 1) but consider these comparisons less reliable, owing to the stronger potential bias caused by different array coverage. Using the >10-kb cut-off, the overall rate of *de novo* CNVs in BD was higher than the rate in controls (4.3 versus 2.0%, *P* = 0.00065) and lower than in SZ (4.3 versus 5.9%, *P* = 0.14).

**Table 1.** *De novo* CNVs detected in this study

Diagnosis	Cytoband	Chr	Start	End	Sample_ID	CNV type	CNV size (bp)	Family_type	FH	Genes
BD	2q31.1	2	175365518	175813657	6038-3	del	448 139	UK BD Sibling	Sibling	*WIPF1*, *CHRNA1*, *CHN1*
BD	5q15	5	92414916	95724985	1702-1	dupl	3 310 069	BG BD Trio	No	18 genes
BD	6p22.3	6	23671880	23724469	6008-1	dupl	52 589	UK BD Trio	No	0
BD	9p21.3	9	23765046	23953634	6194-1	del	188 588	UK BD Trio	No	*ELAVL2*
BD	10p14	10	6968905	6985007	1421-1	dupl	16 102	BG BD Trio	Father	0
BD	10q21.1	10	56517409	56677171	602-1	del	159 762	UK BD Trio	No	*PCDH15*
BD	11q14.1	11	84139937	84345829	6320-1	del	205 892	UK BD Trio	No	*DLG2*
BD	16p13.3	16	6891681	6922307	6198-1	del	30 626	UK BD Trio	No	*RBFOX1*, intronic
BD	16p11.2 distal	16	28825605	29043450	3128-1	dupl	217 845	BG BD Trio	No	12genes
BD	16p11.2	16	29595483	30198151	6023-9	dupl	602 668	UK BD Trio	Father	31 genes
BD	17q23.1	17	57696973	57779678	1002-1	del	82 705	BG BD Trio	No	*CLTC, PTRH2*
BD	18q12.1	18	28277082	28375949	6194-1	dupl	98 867	UK BD Trio	No	0
BD	19q12-q13.12	19	30861683	36685690	2283-1	dupl	5 824 007	BG BD Trio	No	99 genes
BD	20p12.1	20	14771194	14853050	4052-1	del	81 856	BG BD Sibling	sibling	*MACROD2,* exonic
BD	22q11.21	22	21069073	21608479	5003-1	del	539 406	BG BD Trio	Mother	14 genes
SZ	1q41	1	222152402	223209450	UK526-8	del	1 057 048	UK SZ Sibling	Sibling	6 genes
SZ	2p16.3	2	50865334	51492973	4097-6	del	627 639	BG mixed	Sibling	*NRXN1*, exonic
SZ	15q24.3	15	77282884	77340328	UK516-4	dupl	57 444	UK SZ Sibling	Sibling	*PSTPIP1, TSPAN3*
SZ	22q11.21	22	18886915	21463730	UK1238-4	del	2 576 815	UK SZ Trio	No	VCFS region
SZ	22q11.22	22	22698552	23070912	UK602-4	del	372 360	UK SZ Sibling	No	5 genes
SZ	22q11.22 distal	22	22998337	23651318	UK662-4	del	652 981	UK SZ Sibling	Sibling	5 genes

All 21 *de novo* CNVs discovered and validated are sorted by diagnosis and genomic location. Cytoband, chromosome, start and end are listed according to UCSC build 37, hg19. BG, Bulgaria, UK, United Kingdom.

**Table 2.** Comparison of rates and sizes of CNVs with previous *de novo* CNV studies

	*N* trios	*N* CNVs (%) >10 kb	Median size in kb >10 kb	*N* CNVs (%) >100 kb	Median size in kb >100 kb
Current study					
BD	368	15 (4.1%)	189	9 (2.4%)	448
SZ	76	6 (7.9%)	640	5 (6.4%)	653
Malhotra *et al*. (21)					
BD	185	10 (5.4%)	137	5 (2.7%)	611
SZ	177	9 (5.1%)	348	6 (3.4%)	824
CON	426	4 (0.9%)	41	1 (0.2%)	1425
Kirov *et al.* (24)					
SZ	662	34 (5.1%)	321	25 (3.8%)	574
CON	2623	59 (2.2%)	259	46 (1.8%)	320
Xu *et al*. (25)					
SZ	200	17 (8.5%)	260	12 (7.9%)	489
CON	159	2 (1.3%)	2804	2 (1.3%)	2804
Noor *et al*. (23)					
BD	215	8 (3.7%)	76	3 (1.4%)	418
Totals/*P*-value					
BD	768	33 (4.3%)	133	17 (2.2%)	448
SZ	1115	66 (5.9%)	356	48 (4.3%)	613
CON	3208	65 (2.0%)	259	49 (1.5%)	338
*P*-value, BD versus CON		**0.00065**	0.14	0.21	0.74
*P*-value, SZ versus CON		**1.4 × 10^−9^**	0.54	**4.3 × 10^−7^**	**0.001**
*P*-value, BD versus SZ		0.14	0.079	**0.015**	0.44

All rates refer to the number of CNVs in the sample (rather than the number of carriers of CNVs). CNVs on the X-chromosome are excluded.

Significant results are shown in bold.

The median size of *de novo* CNVs was 189 kb in BD and 640 kb in SZ, but this difference was not significant (Mann–Whitney *U*-test, *P* = 0.12). When results were combined with those from previous studies, and a threshold of >100 kb was applied as mentioned above, CNVs in BD were intermediate in size between controls and SZ with medians of 338, 448 and 613 kb in controls, BD and SZ, respectively. Only the SZ/controls difference was significant (*P* = 0.001). The trend for SZ patients to have larger *de novo* CNVs is also clear from the Kaplan–Meier survival graph in Supplementary Material, Figure S1.

Unexpectedly, the rate of *de novo* CNVs was higher in familial cases compared with sporadic ones, both in BD (10.0 versus 3.1%, *P* = 0.039) and in SZ (10.8 versus 5.1%, *P* = 0.42). When we combined our results with the previous ones, the rates of *de novo* CNVs were not significantly different between familial and sporadic cases: for SZ: 5.2 versus 5.9%, *P* = 0.87; and for BD: 6.4 versus 3.3%, *P* = 0.16 (Supplementary Material, Table S2). There was a non-significant trend for parents of *de novo* BD carriers to have been older at the time of birth of their offspring: average paternal age = 31.3 years (SD = 7.4) versus 28.8 (SD = 5.5), *P* = 0.1, and average maternal age = 27.8 (SD = 6.6) versus 25.6 (SD = 5.2), *P* = 0.13. A small trend in the same direction has also been reported in a larger CNV study on probands with intellectual disability (26).

To provide a further comparison of CNVs between BD and SZ, we assessed how many CNVs (transmitted or *de novo*) were found in 15 CNV regions previously implicated in SZ (12) in the BD and SZ probands in the current study (i.e. in samples not used in the discovery of these associations). The results are presented in Table 3. The overall rate of these CNVs in BD is significantly lower (1.35%) than that in the SZ sample (9%) (Fisher Exact test, *P* = 0.0007), and on six occasions, they were not transmitted to BD probands from carrier parents. In contrast, there were no non-transmitted CNVs from this list in the SZ sample. No person had two CNVs from this list.

**Table 3.** Transmission status of CNVs at loci implicated in SZ [according to our review of the literature (12)]

Locus	Position in Mb	BD (*N* = 371)	SZ (*N* = 78)
1q21.1 del	chr1: 14657–14739	1 T from BD F	
1q21.1 dup	chr1: 14657–14739	1 T from healthy M	
*NRXN1* del	chr2: 5015–5126		1 *de novo*
3q29 del	chr3: 19573–19734		
WBS dup	chr7: 7274–7414		
*VIPR2* dup	chr7: 15882–15894		
15q11.2 del	chr15: 2280–2309	3 NT from healthy parents	5 T (one M is SZ)
Angelman/Prader–Willi dup	chr15: 2482–2843		
15q13.3 del	chr15: 3113–3248		
16p13.11 dup	chr16: 1551–1630	1 T 2 NT from healthy parents	
16p11.2 distal del	chr16: 2882–2905		
16p11.2 dup	chr16: 2964–3020	1 *de novo* 1 NT from healthy M	
17p12 del	chr17: 1416–1543	1 T from healthy F	
17q12 del	chr17: 3481–3620		
22q11.2 del	chr22: 1902–2026		1 *de novo*
Total in probands		5 (1.35%); 6 NT	7 (9%); 0 NT

The ‘Total in probands’ include transmissions + *de novos*.

T/NT, transmitted/not transmitted from a parent; M, mother; F, father.

**Figure 2.**
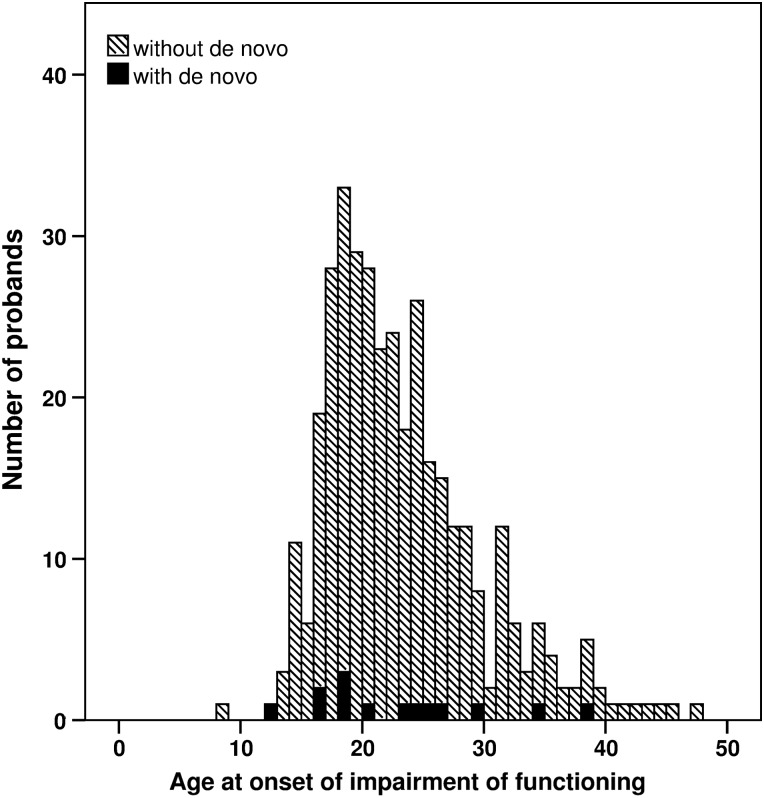
Age at onset among BD probands with *de novo* CNVs (black columns) and without *de novos* (dashed columns).

One previous study on BD found an increased rate of singleton deletions in subjects with an onset of illness before the age of 18 years (17). Another one (21) found an increased rate of *de novo* CNVs in early-onset BD cases. In the present study, the mean age at onset among BD probands with and without *de novos* was practically identical (22.6 versus 22.8 years), with a similar distribution of ages (Fig. 2).

Gene pathway analyses in the combined datasets did not reveal an enrichment of BD *de novo* CNV hits relative to control *de novo* CNVs after controlling for multiple testing (Supplementary Material).

## DISCUSSION

We have conducted the largest analysis of *de novo* CNVs in BD to date (Table 2). We analysed BD and SZ families together, in order to compare the *de novo* rates with the same methods and arrays.

### Frequency of *de novo* CNVs in BD

*De novo* CNVs were found in 4.1% of BD probands. This rate is increased compared with controls from previous studies, but lower than the 7.9% in SZ in the current sample. To obtain a more meaningful comparison, we included in our analyses data from previous large studies on *de novo* CNVs in SZ, BD and controls. In order to minimise possible bias caused by different array resolutions used in the different studies, we analysed these differences for CNVs >100 kb (as these are more likely to be detected by all arrays). Table 2 and Figure 1 show the rates in these phenotypes in the combined data. The rate in BD probands was intermediate between those in controls and SZ (1.5 versus 2.2 versus 4.3%), although the rates in BD were not significantly different from controls. Despite the weak statistical support, both comparisons (with cut-offs of 10 and 100 kb) show similar trends, with the rates of *de novo* CNVs in BD being intermediate between controls and SZ (Fig. 1).

### Potential role of specific CNVs in BD

The trend we observed for an increased rate of *de novo* CNVs in BD compared with controls is consistent with a small proportion of these loci playing a role in the pathogenesis of the disorder. The more likely candidate loci are the following: the deletion at *DLG2*, a gene implicated in SZ (24) and BD (23); the duplication at 16p11.2, as it is also implicated in SZ and BD (13,21) [our proband with *de novo* duplication was among the cases used in the original case–control study that found an association with BD (13)]; the duplication of the ‘distal 16p11.2’ locus, as it is an ID locus, while the reciprocal deletion is both an SZ and ID locus (15,27); the deletion at *PCDH15*, as mutations in this gene can cause deafness and Usher syndrome Type IF (http://omim.org/entry/602083), a disorder with a possibly increased rate of psychosis and behavioural problems (28,29).

### CNVs in BD might be less pathogenic than those found in SZ

Overall *de novo* CNVs tended to be smaller in BD (median of 448 kb) than in SZ (median of 613 kb) in the combined datasets (Table 2). The lack of significance might be due to the small sample sizes, as the distribution of CNV sizes suggests a trend for CNVs in SZ to be larger (Supplementary Material, Fig. S1). Previous case–control studies in BD also report that the rate of very large (>1 Mb) and rare (<1%) CNVs in BD is similar or even lower than that in controls (18,22). Only two deletions and six duplications in BD probands in the current study were >1 Mb (rates of 0.54 and 1.62%, respectively, including transmitted CNVs). These rates are lower than those in previous controls analysed by us with the same methods: among 11 255 controls in our recent study, we reported rates of 0.65 and 1.95%, respectively (30). A smaller proportion of CNVs in BD probands were also found at 15 loci that have been shown to be pathogenic for SZ and other neurodevelopmental disorders, either as *de novo*, or inherited (Table 3). The cumulative rate of 1.35% of these CNVs in BD patients is close to the 0.96% rate we reported among 11 255 controls and lower than the 2.49% among 6882 SZ patients in our recent study (30). The strongest difference between the two disorders is for 15q11.2 deletions, which were not transmitted from three unaffected parents to their BD offspring, whereas such deletions were transmitted from three parents (one affected with SZ) to five SZ offspring (including two affected SZ sib-pairs). In our previous study on BD, we also found a particularly low rate of 15q11.2 deletions among 1697 cases (0.18%) (18), which is even lower than the 0.28% in population controls (12). All these observations suggest that very large and rare CNVs, and those shown to increase risk for SZ, ID and DD, play at best a very modest role in BD.

### Cases with a positive family history also have an increased rate of *de novo* CNVs

It has generally been assumed that sporadic cases are more likely to carry *de novo* CNVs than familial cases. To test whether *de novo* CNVs are more common in sporadic cases, we stratified the sample by history of BD/SZ/psychotic disorder in first-degree relatives and found that the *de novo* CNV rate is not significantly different between familial and sporadic cases; in fact, it was higher in familial cases in the current study (Supplementary Material, Table S2). The first study of *de novo* CNVs in SZ reported that they occur more frequently in sporadic cases (25), but a subsequent study (21) found that the rates of *de novo* CNVs in BD and SZ cohorts were similar in sporadic and familial cases. In our previous study on SZ (24), we considered the family history as positive only if it was present in parents (reasoning that siblings can have independent *de novo* CNVs) and found a slightly higher rate in sporadic cases. Combining all these studies and re-coding our previous data (24) to include cases with affected siblings as well, we find that the rate of *de novo* CNVs is similar in familial and sporadic cases: 5.2 versus 5.9% for SZ and 6.4 versus 3.3% for BD, respectively (the increased rate in familial BD cases is not significant, *P* = 0.16).

Two examples of *de novo* CNVs in family history-positive cases are particularly striking. The 16p11.2 duplication has a high penetrance of 34% for any neurodevelopmental disorder (31) but was found in the daughter of a father who also suffers with a severe form of BD (being *de novo*, that mutation is not found in the father). The exonic *NRXN1* deletion [penetrance of 32% for any disorder (31)] was found in a SZ proband from a multiply affected family: the proband's sister had schizoaffective disorder, she was married to a BD patient and her daughter had BD. No other family member had a pathogenic CNV.

In conclusion, this study confirms previous suggestions that very large and rare CNVs, especially those implicated in neurodevelopmental disorders (such as most of the CNVs listed in Table 3), play a lesser role in BD compared with SZ. However, we did observe a non-significant trend for the rate of *de novo* CNVs to be higher in BD than in controls, suggesting that larger and more powerful studies might reveal a significant excess. In addition, several of the loci impacted by *de novo* CNVs have been previously implicated in neuropsychiatric disorders, which enhance their credibility as candidates for BD. These include 16p11.2, *DLG2* and *PCDH15*. Finally, we also observed an excess of *de novo* mutations in familial cases of major psychiatric disorders. With hindsight this should not be surprising, as disorders of complex genetic inheritance are not due to single gene defects, but to an accumulation of a number of susceptibility factors.

## MATERIALS AND METHODS

### Participants

The total sample (after QC) filtering consists of 449 probands: 368 with BD (256 from Bulgaria and 112 from the UK) and 76 with SZ (15 from Bulgaria and 61 from the UK). Three hundred and eighty-one probands were from parent-offspring trios (342 with BD and 39 with SZ), 42 were from families with 2 affected siblings (16 with BD and 26 with SZ) and 21 (10 BD and 11 SZ) were from families with more complex structures, including families with a mixture of diagnoses (Supplementary Material, Table S1). Probands affected with schizoaffective disorder were excluded from this study. Probands with a history of psychosis in a sibling or parent (50 with BD and 37 with SZ) were included, as none of the risk CNVs identified to date is sufficiently penetrant to fully explain the disorder in carriers (31), and therefore, we wanted to test whether familial cases can also have *de novo* CNVs. The proportion of affected sibling pairs with SZ from the UK is very high because part of this cohort was recruited as affected sib-pairs for linkage analysis, whereas all BD trios were recruited specifically for studying parent-offspring trios.

The recruitment of families in Bulgaria has been described before (24). Each proband had a history of hospitalisation and was interviewed with an abbreviated version of the Schedules for Clinical Assessment in Neuropsychiatry (SCAN) (32). Consensus best-estimate diagnoses were made according to DSM-IV criteria by two researchers. This recruitment also included SZ trios, which have been genotyped with Affymetrix arrays and reported previously (24), apart from some families with different diagnoses that are reported here. In the UK, the BD patients were recruited and interviewed in person by GK, using the same rating instruments. Consensus best-estimate diagnoses were made by two researchers (G.K. and N.C.), based on the interview and hospital notes. The SZ families from the UK were recruited as part of sib-pair and case–control collections. The main purpose for the inclusion of SZ probands in the current study is to compare in an unbiased way (using identical methods), the *de novo* CNV rate between BD and SZ, and also to enlarge the sample of family history-positive cases, where fewer data are available from previous studies. Ethics committee approval for the study was obtained from the relevant research ethics committees and all individuals provided written informed consent for participation. A small proportion of the probands from the UK have been included as cases in previous case–control studies: 55 BD probands are in the Grozeva *et al*. study (18) and 29 SZ probands in the Kirov *et al*. study (33); however, they were not evaluated for *de novo* CNVs. Comparisons with d*e novo* CNVs from healthy control populations were made with probands from three previous studies (21,24,25).

Genotyping of blood-derived DNA from all samples was performed at the Stanley Centre for Psychiatric Research at the Broad Institute of MIT, USA on two arrays: HumanOmniExpress-12v1 (referred further for short as ‘OmniExpress array’), containing 730 525 probes, and any poorly performing samples were re-genotyped on HumanOmniExpressExome-8v1 (‘Combo array’), containing 951 117 probes. The Combo array contains SNPs from both the Omni Express array and the Illumina HumanExome-12v1_A (‘Exome array’); however, for the analysis of Combo array data, we only used the probes present on the OmniExpress array (*N* = 699 865).

### CNV calling and QC

Raw intensity data were processed using Illumina Genome Studio software (v2011.1). SNPs were clustered using the current samples, and LRR and B-allele frequencies were generated for CNV detection. PennCNV (34) was used to call CNVs following the standard protocol and adjusting for GC content. Sample-level QC was performed using the QC metrics generated by PennCNV. These include: LRR standard deviation, B-allele frequency drift, wave factor and total number of CNVs called per person. Samples were excluded if for any one of these metrics they constituted an outlier in their source dataset (details not presented). All poorly performing samples were re-genotyped on Combo arrays. If one family member making up a trio was excluded, then we excluded the whole trio, thus excluding 33 families.

All individual CNVs also went through QC filtering. First, raw CNVs in the same sample were joined together if the distance separating them was <50% of their combined length. CNVs were then excluded if they were either <10 kb, covered by <10 probes, overlapped with low copy repeats by >50% of their length (using PLINK) (35) or had a probe density of >20 kb per probe. The remaining CNVs from each dataset were then analysed together and CNV loci with a frequency of >1% were excluded using PLINK. The putative *de novo* CNVs were validated with the median z-score outlier method (24), software freely available at http://x004.psycm.uwcm.ac.uk/~dobril/z_scores_cnvs. The z-score histograms of CNVs with marginal z-scores were manually inspected. For all putative *de novo* CNVs, the LRR and B-allele frequencies were also visually inspected using Illumina Genome Studiov2011.1 software. Validation of all remaining putative *de novo* CNVs was performed using real-time PCR based on SYBR-Green I fluorescence with at least three primer sets per CNV. All samples were amplified using Sensimix kit (Bioline, UK), and data were analysed using Rotor-Gene Q series software. Each primer set was compared with a primer set outside the CNV which served as ‘control’ and data were normalized using delta Ct (cycle threshold) values.

## SUPPLEMENTARY MATERIAL

Supplementary Material is available at *HMG* online.

## FUNDING

The work at Cardiff University was funded by Medical Research Council (MRC) Centre (G0800509) and Program Grants (G0801418) and an MRC PhD Studentship to E.R. Funding for the recruitment of trios in Bulgaria was provided by the Janssen Research Foundation in 1999–2004, Ref: 045856. Funding for the recruitment of BD samples in the UK was provided by the Wellcome Trust as a Training Fellowship to G.K. in 1996–1999, Ref: 045856. The samples were genotyped at the Broad Institute, USA, funded by a philanthropic gift to the Stanley Center for Psychiatric Research. Funding to pay the Open Access publication charges for this article was provided by Research Councils UK.

## Supplementary Material

Supplementary Data
