# Supplementary material for: De novo CNVs in bipolar affective disorder and schizophrenia
Source: Hum Mol Genet. 2014 Jul 23;23(24):6677–83. doi: 10.1093/hmg/ddu379 (PMC4240207; doi:10.1093/hmg/ddu379)
Supplement: Supplementary Data [file supp_23_24_6677__index.html]

De novo CNVs in Bipolar Affective Disorder and Schizophrenia — De novo CNVs in bipolar affective disorder and schizophrenia — De novo CNVs in bipolar affective disorder and schizophrenia — Supplementary Data 

# *De novo* CNVs in bipolar affective disorder and schizophrenia

## Supplementary Data

Supplementary Data

**Files in this Data Supplement:**

- Supplementary Data - Docx file
