# Supplementary material for: De novo CNVs in bipolar affective disorder and schizophrenia
Source: Hum Mol Genet. 2014 Jul 23;23(24):6677–83. doi: 10.1093/hmg/ddu379 (PMC4240207; doi:10.1093/hmg/ddu379)
Supplement: Supplementary Data [file supp_ddu379_ddu379supp.docx]

**Supplementary material to “*De novo* CNVs in Bipolar Affective Disorder and Schizophrenia”**

|  | **Bulgaria** | | **UK** | |
| --- | --- | --- | --- | --- |
| **Family type** | **BD**  **probands** | **SZ**  **probands** | **BP**  **probands** | **SZ**  **probands** |
| Trio | 242 | 4 | 100 | 35 |
| Probands with affected siblings | 8 | 0 | 8 | 26 |
| Probands in large or mixed families | 6 | 11 | 4 | 0 |
| Total | 256 | 15 | 112 | 61 |
| Probands with negative family history | 230 | 7 | 88 | 32 |
| Probands with positive family history | 26 | 8 | 24 | 29 |

**Table S1**. Numbers of probands analysed in the current study. The numbers given in the table are those of patients who had both parents genotyped. The number of families is lower, e.g. a family with two affected children contributes two counts to the table. “Mixed” families refer to families with cases of both BD, SZ or schizoaffective disorder. The bottom two rows indicate the number of probands with negative or positive family histories of BD/SZ/schizoaffective disorder in a first-degree relative.

|  | **This study** | | **Xu et al, 2008 ^3^** | | **Kirov et al 2012 ^2^** | | **Malhotra et al 2011 ^1^** | | **Totals** | |
| --- | --- | --- | --- | --- | --- | --- | --- | --- | --- | --- |
|  | **N probands** | **N**  **CNVs** | **N probands** | **N**  **CNVs** | **N probands** | **N**  **CNVs** | **N probands** | **N**  **CNVs** | **N probands** | **N**  **CNVs** |
| Sporadic SZ | 39 | 2 (5.1%) | 152 | 17 (11.2%) | 571 | 30 (5.3%) | 97 | 4 (4.2%) | 859 | 51 (5.9%) |
| Familial SZ | 37 | 4 (10.8%) | 48 | 0 | 91 | 4 (4.4%) | 44 | 3 (6.8%) | 210 | 11 (5.2%) |
| Sporadic BD | 318 | 10 (3.1%) |  |  |  |  | 78 | 3 (3.8%) | 396 | 13 (3.3%) |
| Familial BD | 50 | 5 (10.0%) |  |  |  |  | 107 | 5 (4.7%) | 157 | 10 (6.4%) |

**Table S2**. Rates of *de novo* CNVs in sporadic and familial cases: comparison with previous studies. In the Kirov et al (2012) paper we had reported the rate on families with an affected parent only and found only 1.6% rate in such cases. We have now re-analysed these results, recoding cases with affected siblings to have positive family history, in line with the current study.


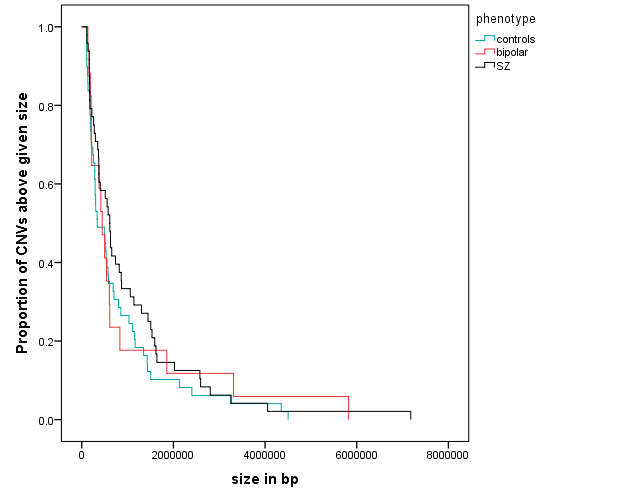


**Figure S1.** Size of copy number variants (CNVs). Kaplan–Meier survival graph for the size of de novo CNVs in BD cases, SZ cases and controls based on all studies listed in Table 2. CNVs of <100kb are filtered out, to improve comparison between studies.

**Gene pathway analysis:** Tests of gene pathways enrichment for case *de novo* CNV hits relative to control *de* *novo* CNV hits from the combined BD and control data from the current and previous studies (1-3) were carried out as described before (2). Briefly, a logistic regression was performed of the case/control status of each CNV on whether that CNV hits a pathway gene, correcting for the length of the CNV and the number of non-pathway genes hit by that CNV. Two sets of pathways were tested for enrichment of case CNV hits: a) the 15 synapse-related and control pathways analysed in our previous study on SZ (2) and b) a comprehensive set of generic pathways, consisting of 1) Gene Ontology (GO) (4) (http://www.geneontology.org/; accessed 26/7/2013); 2) Kyoto Encyclopedia of Genes and Genomes (KEGG) (5) (http://www.genome.jp/kegg/; accessed 27/6/2011); 3) PANTHER (Protein ANalysis THrough Evolutionary Relationships) pathways version 3.1 (6), accessed February 2012; 4) Mouse Genome Informatics (MGI) database (7), accessed August 2013; 5) REACTOME (8), accessed June 2013; 6) BIOCARTA (http://www.biocarta.com/genes/index.asp, accessed June 2013), 7) NCI (9), accessed June 2013.

Gene pathway analyses in the combined datasets did not reveal an enrichment of BD *de novo* CNV hits relative to control *de novo* CNVs after controlling for multiple testing.

**Bibliography:**

1 Malhotra, D., McCarthy, S., Michaelson, J.J., Vacic, V., Burdick, K.E., Yoon, S., Cichon, S., Corvin, A., Gary, S., Gershon, E.S. *et al.* (2011) High frequencies of de novo CNVs in bipolar disorder and schizophrenia. *Neuron*, **72**, 951-963.

2 Kirov, G., Pocklington, A.J., Holmans, P., Ivanov, D., Ikeda, M., Ruderfer, D., Moran, J., Chambert, K., Toncheva, D., Georgieva, L. *et al.* (2012) De novo CNV analysis implicates specific abnormalities of postsynaptic signalling complexes in the pathogenesis of schizophrenia. *Mol Psychiatry*, **17**, 142-153.

3 Xu, B., Roos, J.L., Levy, S., van Rensburg, E.J., Gogos, J.A. and Karayiorgou, M. (2008) Strong association of de novo copy number mutations with sporadic schizophrenia. *Nat Genet*, **40**, 880-885.

4 Harris, M.A., Clark, J., Ireland, A., Lomax, J., Ashburner, M., Foulger, R., Eilbeck, K., Lewis, S., Marshall, B., Mungall, C. *et al.* (2004) The Gene Ontology (GO) database and informatics resource. *Nucleic Acids Res*, **32**, D258-261.

5 Kanehisa, M., Goto, S., Sato, Y., Furumichi, M. and Tanabe, M. (2012) KEGG for integration and interpretation of large-scale molecular data sets. *Nucleic Acids Res*, **40**, D109-114.

6 Mi, H., Muruganujan, A. and Thomas, P.D. (2013) PANTHER in 2013: modeling the evolution of gene function, and other gene attributes, in the context of phylogenetic trees. *Nucleic Acids Res*, **41**, D377-386.

7 Bult, C.J., Eppig, J.T., Kadin, J.A., Richardson, J.E., Blake, J.A. and Group, M.G.D. (2008) The Mouse Genome Database (MGD): mouse biology and model systems. *Nucleic Acids Res*, **36**, D724-728.

8 Croft, D., O'Kelly, G., Wu, G., Haw, R., Gillespie, M., Matthews, L., Caudy, M., Garapati, P., Gopinath, G., Jassal, B. *et al.* (2011) Reactome: a database of reactions, pathways and biological processes. *Nucleic Acids Res*, **39**, D691-697.

9 Schaefer, C.F., Anthony, K., Krupa, S., Buchoff, J., Day, M., Hannay, T. and Buetow, K.H. (2009) PID: the Pathway Interaction Database. *Nucleic Acids Res*, **37**, D674-679.
